# Observation of natural flexural pulse waves in retinal and carotid arteries for wall elasticity estimation

**DOI:** 10.1126/sciadv.adf1783

**Published:** 2023-06-21

**Authors:** Gabrielle Laloy-Borgna, Léo Puyo, Hidero Nishino, Michael Atlan, Stefan Catheline

**Affiliations:** ^1^ LabTAU, INSERM, Centre Léon Bérard, Université Lyon 1, Univ Lyon, F-69003, Lyon, France.; ^2^ Institute of Biomedical Optics, University of Lübeck, Peter-Monnik-Weg 4, 23562 Lübeck, Germany.; ^3^ Department Science and Technology, Tokushima University, 770-8506, Tokushima, Japan.; ^4^ Centre Hospitalier National d'Ophtalmologie des Quinze-Vingts, INSERM-DHOS CIC 1423, 28 rue de Charenton, 75012 Paris, France.

## Abstract

The risk of cardiovascular events is linked to arterial elasticity that can be estimated from the pulse wave velocity. This symmetric wave velocity is related to the wall elasticity through the Moens-Korteweg equation. However, ultrasound imaging techniques need improved accuracy, and optical measurements on retinal arteries produce inconsistent results. Here, we report the first observation of an antisymmetric pulse wave: the flexural pulse wave. An optical system performs in vivo wave velocity measurements on retinal arteries and veins. Velocity estimation ranges between 1 and 10 millimeter per second. The theory of guided waves confirms the existence of this wave mode and its low velocity. Natural flexural waves can also be detected at the bigger scale of a carotid artery using ultrafast ultrasound imaging. This second natural pulse wave has great potential of becoming a biomarker of blood vessel aging.

## Teaser

A “second” natural pulse wave that could become a biomarker of cardiovascular health has been found.

## INTRODUCTION

Arterial stiffness is an indicator of cardiovascular health and can even be a predictor for cardiovascular mortality (*1*–*5*). This stiffness can be noninvasively estimated by measuring pulse wave velocity (PWV). Known since 1808 thanks to the seminal work of T. Young, the pulse wave is the result of the interaction between stroke volume and artery resistance. As later shown by Moens-Korteweg (1878), the pulse wave propagates at a velocity related to arterial wall stiffness. The existence of two distinct pulse waves was observed for the first time. Similar to compression and shear waves, the coexistence of the standard longitudinal pulse wave (LPW) with an unexpected flexural pulse wave (FPW) is described here.

PWV is most frequently measured on the macrovasculature, which consists of large arteries such as the aorta or the carotid arteries. Now, an increase in aortic PWV is known to be correlated with coronary artery disease (*6*), myocardial infarction (*7*), heart failure (*8*), mortal stroke (*9*), and hypertension (*1*). It has even been indicated to be an independent predictor for cardiovascular events (*3*, *10*). The aortic PWV is sometimes estimated by separately measuring two parameters. First, the time lag between the Doppler waveforms on the common carotid and the right femoral artery is transcutaneously measured (*1*, *3*). Second, the distance covered by the wave is evaluated inaccurately on the surface of the body using a ruler. The ratio between the distance and the time lag produces an aortic PWV estimation. Ultrasound elastography has been used to assess arterial stiffness more accurately, either through the natural PWV (*11*, *12*) or via antisymmetric guided waves generated by an acoustic radiation force applied on a single arterial wall (*13*–*15*). These methods are promising despite the PWV being large compared to the field of view and the frame rate, which limits the accuracy of the measurement.

There are very few methods to estimate arterial stiffness on the microvasculature. Measuring the arterial stiffness at this scale could provide valuable insights for early detection of cardiovascular diseases. Most cardiovascular events are the consequence of a progressive vascular disease called atherosclerosis. It is due to endothelial dysfunction and results in structural remodeling, which has a higher impact on the microcirculation than on large arteries (*16*). The eye, and more precisely the retinal vascular system, is a window that enables noninvasive measurements on the microcirculation system. Furthermore, this system is known to be anatomically correlated with the vasculature of the brain (*17*). As a result, modifications of the retinal vascular system are often associated to stroke-related changes in the cerebrovascular system (*18*). To this day, only a 2020 study by Rezaeian *et al.* (*19*) showed that retinal PWV is positively correlated to carotid-femoral PWV and thus to cardiovascular health. However, PWV measurement on retinal arteries may open a window for early-stage detection strategies of cardiovascular and cerebrovascular pathologies.

Measurements of the retinal PWV reported in literature fluctuate considerably. A 2013 study by Kotliar *et al.* (*20*, *21*) used a method based on dynamic vessel analyzer to find a velocity of 0.4 mm/s for normotensive subjects [they found 21 mm/s in another study (*22*) but corrected this value]. More recently, in 2021, Bedggood *et al.* (*23*) measured 6.4 mm/s using high spatiotemporal resolution adaptive optics. A 2018 study by Li *et al.* (*24*) found, using Doppler optical coherence tomography (OCT), a retinal PWV between 20 and 30 mm/s on young normotensive subjects. Last, Spahr *et al.* (*25*) measured the retinal PWV to be 620 mm/s in their 2015 study using high-speed phase-sensitive spectral domain OCT. Thus, the reported values for the retinal PWV on young normotensive subjects vary by three orders of magnitude.

## RESULTS

### Optical measurements on retinal arteries

**Fig. 1.**
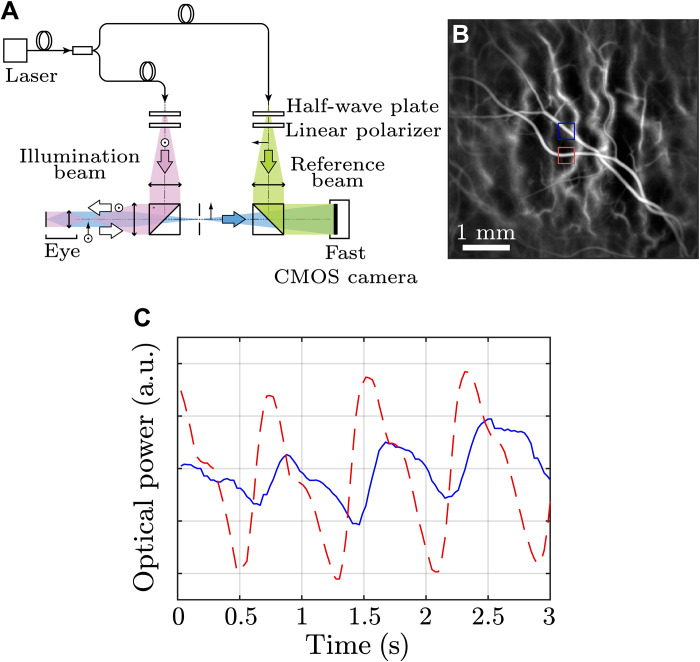
Experimental design. (**A**) Experimental setup of LDH used to acquire blood flow movies in retinal vessels. (**B**) Temporal average of a blood flow movie. The pixel intensity corresponds to the power Doppler signal. (**C**) Temporal evolution of the power Doppler signals in an artery (red) and a vein (blue). Movie S1 was used to get this figure. a.u., arbitrary unit; CMOS, complementary metal-oxide semiconductor.

Retinal blood flow in healthy human subjects was measured using the laser Doppler holography (LDH) technique (*26*–*28*). It consisted in the interferometric and full-field detection of light backscattered by the retina [optical setup presented in Fig. 1A)]. Holograms were digitally reconstructed, and the blood flow was monitored via the intensity fluctuations induced by Doppler-shifted light. Optical power Doppler movies revealing a retinal field of view of 5.3 mm could be obtained with a temporal resolution of 26 ms (38 Hz). Figure 1B presents the temporal average of a Doppler movie obtained from a volunteer. The pixel intensity is proportional to the blood flow power. Doppler signals corresponding to an artery and a vein are presented over time in Fig. 1C. The observed temporal drift in the optical power as a function of time is caused by signal processing but does not influence the wave velocity measurements.

Once the LDH movie was acquired, the objective was to measure the velocity of elastic waves propagating along the artery. Noise correlation algorithms inspired by seismology and applied by Catheline *et al.* for passive elastography (*29*–*32*) were adapted for this particular application. These algorithms are based on spatiotemporal correlation and are influenced by time reversal. Hence, there is no need to control the wave source, thus making these algorithms well adapted to natural waves. From an arbitrary diffuse shear wave field, correlation algorithms allow for measurements of the central wavelength, frequency, and shear wave velocity *c*_T_. Using the elastography assumption, the shear modulus μ is given by the following relationship: $\mu =\rho c_{T}^{2}$, where μ is the shear modulus and ρ the solid density. *c*_T_ is estimated using noise correlation algorithms.

**Fig. 2.**
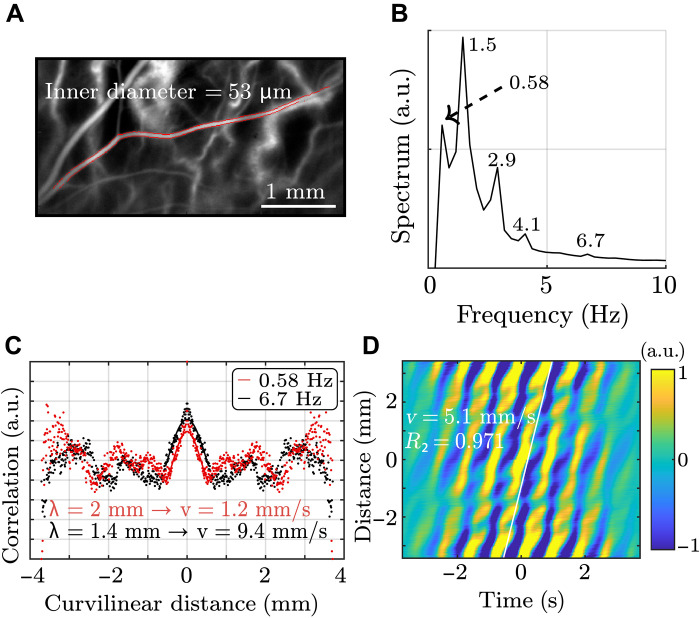
Measurement of the antisymmetric wave velocity in a retinal artery. (**A**) Segmentation of the averaged LDH image. Red points correspond to the edges of the chosen artery. (**B**) Averaged spectrum of the power Doppler signal in an artery. (**C**) Monochromatic focal spots at 0.58 and 6.7 Hz. The points correspond to experimental measurements, and the curves to the sinusoidal function fitted on the central peak. At 0.58 Hz, the measured wavelength was 2 mm, corresponding to a wave velocity of 1.2 mm/s. At 6.7 Hz, the wavelength was 1.4 mm and the velocity is 9.4 mm/s. (**D**) Correlation rate as a function of distance and time is shown. The slope corresponds to the wave velocity, which was measured to be 5.09 mm/s. Movie S2 was used to get this figure.

In the present optical experiments, correlation algorithms were applied along one dimension: the curvilinear coordinate defined by the median line of the artery. To do so, the image acquired using LDH was segmented by an edge detection algorithm based on gradients (MATLAB function called “edge”, with the Sobel approximation). An example of this segmentation is shown in Fig. 2A, where the edges of the blood vessel are delineated in red and superimposed on the holography black and white image. Then, the curvilinear coordinate was defined, and the artery cross sections were identified. Next, the Doppler signal average was calculated for each cross section so as to associate one single time-dependent Doppler signal Φ_x_(*t*) to each abscissa *x*. The mean spectrum of the Doppler signal is presented in Fig. 2B. It comprises several peaks, which can be interpreted as harmonics or subharmonics of the 1.5-Hz fundamental frequency (heart rate). Inspired by noise correlation algorithms, passive spectroscopy algorithms were developed to measure the wavelength at a single frequency rather than the global group velocity. Monochromatic focal spots at frequency *f* could be calculated from the Fourier transform of the Doppler signal $\hat{\phi_{s}}(f)$ as a function of the curvilinear coordinate *r*

$$C(f,r)={\left\langle \cos\{\arg[\hat{\phi_{s}}(f)]-\arg[\hat{\phi_{s+r}}(f)]\}\right\rangle }_{s}$$ (1)

The monochromatic focal spots were calculated for each spectrum peak. Figure 2C shows two examples at 0.58 and 6.7 Hz. The elastic wavelength was measured using a sinusoidal fit around the central peak. At 0.58 Hz, the measured wavelength was 2 ± 0.05 mm, corresponding to a phase velocity of 1.16 ± 0.03 mm/s at this frequency. Similarly, the measured wavelength at 6.7 Hz was 1.4 ± 0.04 mm, corresponding to a wave velocity of 9.4 ± 0.3 mm/s. A measurement at 1.5 Hz was conducted on five different arteries of the same volunteer, and the average velocity was measured to be 4.2 ± 1.8 mm/s. These velocity values cannot be associated with bulk shear waves (*33*) because that would correspond to a shear modulus of approximately 1 Pa, which is not physically consistent. Moreover, these measured wave velocities are far lower than the PWV values usually found in the carotid artery (*11*, *12*, *19*) (at least by a factor of 1000). Their values were found to fall between the measurements reported by Kotliar *et al.* (*21*) (0.4 mm/s) and those reported by Li *et al.* (*24*) (20 to 30 mm/s) for the retinal PWV. A more classical time-of-flight technique was also tested to verify these measurements. It simply consisted in following the wavefront over time. The result of this independent measurement is shown in Fig. 2D. A spatiotemporal representation of the Doppler signal was used, thus allowing the observation of stripes for which the slope corresponds to the wave velocity. A value of 5.09 ± 0.03 mm/s was found, which is compatible with the previously mentioned measurements. Therefore, the wave velocity values found using two independent methods were consistent with one another and ranged between 1 and 10 mm/s depending on the measurement frequency.

### Comparison with existing theories

The expression for the PWV was derived by Moens-Korteweg at the end of the 19th century (*34*, *35*), which was later discussed (*36*, *37*) and accepted by the community. By considering the propagation of an axisymmetric wave along a liquid-filled tube, it states that

$$\mathrm{PWV}=\sqrt{\frac{h}{d}}\sqrt{3}c_{T}$$ (2)

where *h* is the wall thickness, *d* the vessel diameter, and $c_{T}=\sqrt{\frac{\mu }{\rho }}$ is the bulk shear wave velocity. This equation is commonly used to calculate the arterial elasticity from the PWV. It takes into account the effect of blood inside the tube. However, this equation would lead to calculating a shear wave velocity of 5 mm/s, which is far too small to be physically consistent. According to elasticity theory, this axisymmetric pulse wave is only one of two possible guided waves propagating along a tube. Torsional and flexural waves do also exist.

**Fig. 3.**
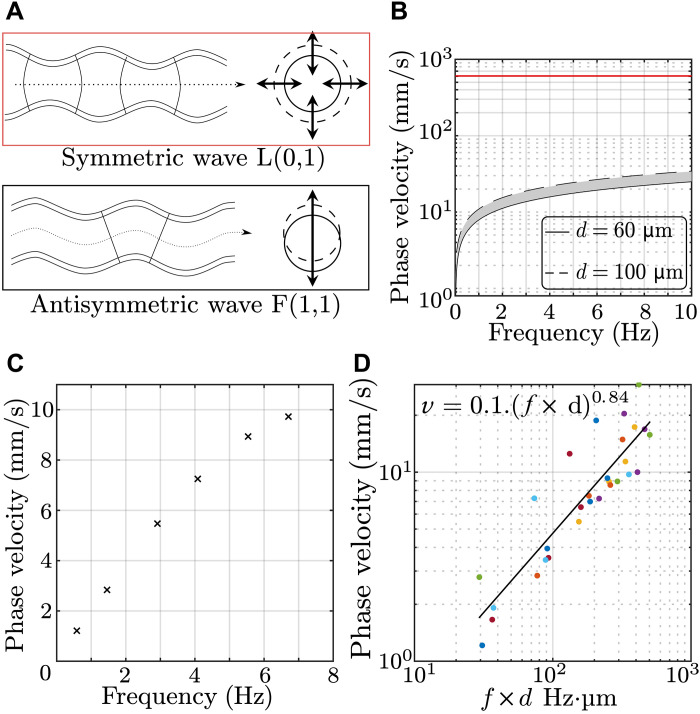
Wave velocity dispersion in retinal arteries. (**A**) Schematics of the deformation associated with the two types of waves propagating along a tube. (**B**) Theoretical dispersion curves for each mode. (**C**) Experimental dispersion curve of the antisymmetric mode obtained in a retinal artery. (**D**) Phase velocity as a function of the product [frequency × diameter] for five arteries of the same volunteer, each identified by a different color. Movies S1 to S4 were used to get this figure.

The equations describing the propagation of guided waves in tubes were derived in 1959 by Gazis *et al.* (*38*, *39*) and numerically computed for nondestructive testing by Nishino *et al.* (*40*) in 2001. Using Gazis’ notations, two propagating waves L(0,1) and F(1,1) correspond to the classical pulse wave and to a flexural wave, respectively. The first one is associated to a diameter expansion having an axisymmetrical deformation, as shown in Fig. 3A. The second one is associated to the tube flexion without any cross-sectional change. This deformation is antisymmetric as shown in Fig. 3A. Note that this flexural wave has to be distinguished from the flexion waves generated by ultrasound radiation pressure and propagating on a single arterial wall (*12*–*14*); in our case, it is a global tube wave and is naturally present in blood vessels. For the rest of this work, the following notations are used: LPW for L(0,1) mode and FPW for F(1,1) mode. In the case of a hollow cylinder made of an elastic and isotropic material surrounded by air, velocity expressions for both modes can be approximated at low frequencies by the equations presented hereunder

$$v_{\mathrm{FPW}}≅\sqrt{\frac{\pi fdc_{T}}{\sqrt{3}}}\;\mathrm{and}\;v_{\mathrm{LPW}}≅\sqrt{3}c_{T}$$ (3)

where *f* corresponds to the frequency. From Eq. 3, it can be noticed that at low frequencies, the LPW velocity expression does not depend on geometrical parameters *h* and *d*, contrary to the Moens-Korteweg equation. The reason is that Gazis did not consider having any liquid inside the tube. Therefore, Gazis’ theory cannot include Moens-Korteweg’s equation for the symmetric wave. However, reciprocally, Moens-Korteweg does not account for the existence of several waves propagating along the artery as described by Gazis. From Eq. 3, it can be observed that, at low frequencies, the FPW velocity can be very low and could explain the low experimental estimations reported in Fig. 2.

Because the FPW is highly dispersive, it is of high interest to study its dispersion: the evolution of wave velocity with respect to frequency. Using passive spectroscopy, the wavelength was measured at each frequency, allowing the retrieval of the phase velocity as a function of frequency, as shown in Fig. 3C. As expected for an antisymmetric plate wave, the phase velocity increases as a function of frequency following a power-law behavior. Therefore, we report here the first observation of this FPW, whose nature was confirmed by the dispersion trend.

To provide a more quantitative comparison between the measurements and the theoretical dispersion predicted by Gazis’ equations, a numerical computation was performed to obtain the dispersion curve across the entire frequency range. The parameters used for this calculation were the following: 60 and 100 μm for the tube diameter, 15 μm for the arterial wall thickness [taken from (*41*)], 1500 m/s for the compression wave velocity (standard ultrasound velocity in water), and 0.35 m/s for the shear wave velocity [to obtain a symmetrical PWV of 600 mm/s as measured by Spahr *et al.* (*25*)]. Note that using the Moens-Korteweg equation while considering a LPW velocity of 620 mm/s, one would obtain 0.8 m/s for the bulk shear wave velocity. However, for the purpose of consistency, Gazis’ equations were used for both wave modes. The corresponding dispersion curves are presented in Fig. 3B. Across the studied frequency range, it is confirmed that the symmetrical pulse wave has a constant and much higher wave velocity than the antisymmetric wave. Moreover, the calculated wave velocity is shown to increase with frequency as it varies from 1 to 25 mm/s when the frequency is increased from 0 to 10 Hz. Hence, the results can greatly differ depending on the measurement frequency. The theoretical and experimental dispersion curves are in good qualitative agreement because both show similar trends. However, there is a difference of a factor of 2 (at least) between both curves.

The phase velocity was measured on five different arteries of the same volunteer and at different frequencies for each artery. These measurements are all presented in Fig. 3D, where the phase velocity is plotted as a function of the product of the frequency and the diameter of the arteries (*f* × *d*). Note that the diameter measured on the LDH movies is not suitable because it corresponds to the inner diameter, whereas the phase velocity should be proportional to the outer diameter. A linear fit on a logarithmic plot shows a power of 0.84 for the evolution of the phase velocity as a function of *f* × *d*. This result does not match the 0.5 power predicted by the theory. The actual case of an artery filled with blood and embedded in a soft medium is not perfectly described by the model of a hollow cylinder surrounded by air. These discrepancies will be further discussed.

### Ultrasound measurements on carotid arteries

**Fig. 4.**
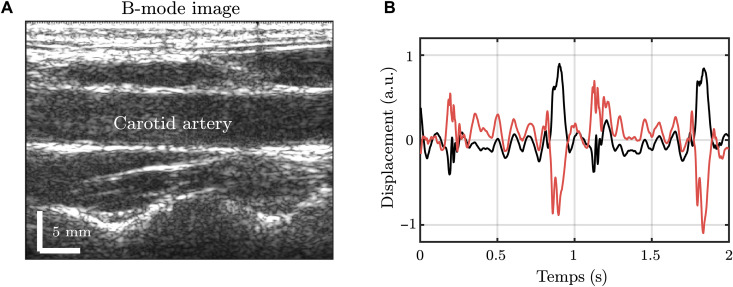
Ultrafast ultrasound experiment in the carotid artery. (**A**) Ultrasound B-mode image of the carotid artery. (**B**) Displacements of one point on the top and bottom walls of the artery, represented in red and black, respectively. Movie S5 was used to get this figure.

**Fig. 5.**
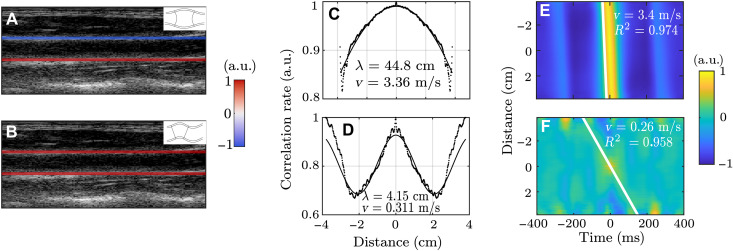
Wave velocity measurements in the carotid artery. B-mode image of the carotid artery superimposed with a snapshot of the displacements of the edges at a time when the displacements are (**A**) symmetric and (**B**) antisymmetric. Focal spots of (**C**) the symmetric wave and (**D**) the antisymmetric wave at 7.5 Hz, which allow for measuring the wavelength and thus the wave velocity at this frequency. Wave velocity measurements using the time-of-flight technique for (**E**) the symmetric wave and (**F**) the antisymmetric wave. Movie S5 was used to get this figure, the symmetric displacement corresponding to time 0.902 s and the antisymmetric one corresponding to 0.608 s.

The existence of flexural waves propagating along an artery has no reason to be limited to retinal arteries. Another experiment was thus conducted on the carotid artery, the most studied artery for PWV because of its accessibility for ultrasound imaging. The experiment consisted of a simple ultrafast ultrasound acquisition to record an image sequence of the carotid artery moving as a result of the propagation of natural pulse waves. A B-mode image obtained from this acquisition is presented in Fig. 4A. Using phase-tracking algorithms, the displacements of the edges of the carotid artery along the ultrasonic beam could be estimated. The displacements of two points taken respectively on the top and bottom edges of the carotid artery are represented with respect to time in Fig. 4B. The heartbeat can be identified on both displacement curves showcasing a symmetric deformation of the carotid artery in time. However, there is also clear evidence of an antisymmetric deformation of the carotid artery because displacement of both edges often exhibits the same displacement sign on both curves. Depending on the chosen timeframe, symmetric or antisymmetric displacements can be observed, indicating that both waves coexist along the artery. An example of the displacement types is shown in Fig. 5 [A (symmetric) and B (antisymmetric)]. This is the first conclusion supported by this experiment: Despite the scale change compared to retinal arteries, both wave types do propagate along the carotid artery.

By calculating either the sum or the difference of both displacements, the antisymmetric or the symmetric waves can be selected, respectively. Figure 5C shows the symmetric wave’s averaged monochromatic focal spot at 7.5 Hz, which allowed the measurement of a wavelength of 44.8 ± 0.2 cm, corresponding to a wave velocity of 3.36 ± 0.01 m/s. This order of magnitude is consistent with what is usually reported for the pulse wave in the carotid artery (*11*, *12*) and with the wave velocity of 3.41 ± 0.02 m/s that was measured using the time-of-flight technique (see Fig. 5E). The same analysis was conducted for the antisymmetric wave (see Fig. 4, D and F), and wave velocities of 0.31 ± 0.01 m/s using passive spectroscopy and 0.256 ± 0.003 m/s using time of flight were obtained. Hence, the flexural wave velocity is much smaller than the axisymmetric wave velocity. This observation was expected, given the results of the numerical computations presented in Fig. 3B. However, it is worth be mentioning that different values were found when estimating the elastic modulus using one or the other wave mode velocity. The shear elastic modulus estimated from velocity measurements should be independent of the wave type in consideration. The difference here is probably due to the assumptions underlying Gazis’ equations that inaccurately describe wave propagation along real arteries.

**Fig. 6.**
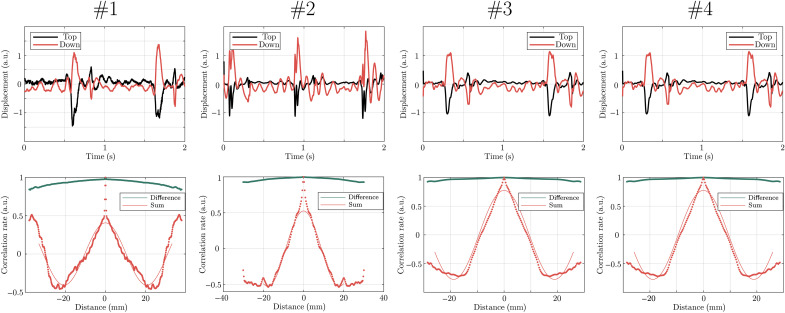
Several experiments conducted on four healthy volunteers. Displacements of one point on the top (black) and bottom (red) edges of the carotid artery over time for each volunteer (top row). Focal spots for the sum (corresponding to the antisymmetric wave) and the difference (corresponding to the symmetric wave) of the displacements of both edges, allowing us to measure the wavelengths and thus the wave velocities of each mode. Volunteers 1 to 4 correspond to movies S6 to S9, respectively.

Furthermore, the experiment was conducted on several healthy volunteers to support the existence of two distinct pulse waves in the carotid artery. For each of these four volunteers, the displacements of one point on the top and bottom edges are represented over time in Fig. 6 (top row). For all volunteers, the carotid deformation during the heartbeat is mainly symmetric. However, there is obvious evidence of an antisymmetric deformation between heartbeats because the displacements of both edges have the same signs. The polychromatic focal spots used to measure central wavelengths for both wave modes are shown in the bottom row of Fig. 6. The axisymmetric wavelength is much larger than the antisymmetric wavelength, leading to much larger wave velocities for the symmetric wave. The central frequencies of both wave modes were measured separately using autocorrelation. They ranged between 6 and 19 Hz. Last, wave velocities were obtained and reported in Table 1. The reproducibility of the experiment was also investigated. To do so, the experiment was conducted five times on volunteer 1, resulting in an average velocity of 3.44 ± 2 m/s for the axisymmetric wave and 0.78 ± 0.1 m/s for the antisymmetric wave. As expected, given the large wavelength differences between the two wave modes, the measurement is more accurate for the antisymmetric pulse wave. A whole wavelength is visible on the 4 cm width of the ultrasonic probe, resulting in a more reliable measurement of wavelength and wave velocity. This should lead to increased precision when estimating shear elasticity.

**Table 1.** Summary of the measurements conducted on four healthy volunteers. For each of the four healthy volunteers, the gender, age, wavelengths, and wave velocities for the symmetric and the antisymmetric pulse waves are reported. The symmetric wave velocity was measured to be between 3.5 and 5 m/s, which is consistent with what is usually reported, while the antisymmetric wave velocity was measured to fall between 0.4 and 0.7 m/s for all volunteers. Volunteers 1 to 4 correspond to movies S6 to S9, respectively.

Volunteer	Gender (M/F)	Age	λ_*L*(0,1)_ (cm)	*v*_*L*(0,1)_ (m/s)	λ_*F*(1,1)_ (cm)	*v*_*F*(1,1)_ (m/s)
1	M	52	46.5 ± 0.2	3.10 ± 0.03	4.13 ± 0.03	0.496 ± 0.007
2	F	27	47.3 ± 0.2	4.88 ± 0.04	3.56 ± 0.05	0.69 ± 0.02
3	M	22	53 ± 1	3.3 ± 0.1	3.77 ± 0.05	0.47 ± 0.01
4	F	26	35.9 ± 0.1	2.25 ± 0.01	4.51 ± 0.05	0.67 ± 0.01

## DISCUSSION

The experimental results in this work showed that at least two pulse waves naturally exist in blood vessels: an axisymmetric (LPW) and an antisymmetric (FPW) pulse waves.

For this study, passive spectroscopy algorithms were developed, allowing to study the dispersion of the waves by measuring their velocities at each frequency. The model of an empty tube embedded in the air does not perfectly describe the real case of a blood vessel yet. This is why the dispersion predicted by the model only partially predicts what is found experimentally. In particular, the blood flowing inside the artery and the surrounding soft tissues influence the propagation of elastic waves. These two phenomena tend to slow down the elastic waves, resulting in an overestimation of the wave velocity by the model. Hence, refinement is needed to accurately measure arterial stiffness. An equivalent of the Moens-Korteweg equation (Eq. 2) is required to link clinical wave speed measurements to elastic properties. This theoretical part is in progress.

The LPW and the FPW exhibit fundamental differences in the deformation geometry. The LPW corresponds to the propagation of an axisymmetric deformation, whereas the FPW is due to an antisymmetric deformation. The experiment conducted on the carotid artery using ultrasound imaging showed that both types of waves exist in this artery and are propagating simultaneously. However, when the heart beats, an overpressure is applied to the artery, inducing an axisymmetric deformation of the vessel. The source symmetry explains why the LPW dominates the FPW when the heart beats. However, at other instants, the symmetric wave has weaker amplitudes, allowing the antisymmetric wave to be detected. For a vein with no pulsatility, there is no symmetric source; hence, there is no LPW propagating. However, the FPW could be detected since the tube can guide any noise motion, resulting in an antisymmetric wave. An example of a flexural wave velocity measurement on a retinal vein is presented in fig. S1, where the same order of magnitude for the wave velocity values is retrieved. This is a major advantage of the FPW discovery: This wave could be used to characterize the elasticity of veins, not only arteries, opening perspectives for clinical applications.

In retinal arteries, the LPW was not detected, and this is due to several reasons. First, the Doppler signal average over a given cross section eliminates the axisymmetric contribution. Second, the noise correlation method used to perform the velocity measurements is more sensitive to large curvatures corresponding to small wavelengths. Therefore, if two waves were superimposed, then the method would preferably measure the slowest wave velocity, the flexural wave. Third, the holographic videos processed have a low frame rate of 38 Hz, which is too low to detect a wave as fast as the axisymmetric pulse wave. To see both wave modes, the same analysis could be conducted on movies with a higher frame rate. Nevertheless, high-speed movies are too noisy to be processed. This feature indicates that antisymmetric wave detection does not require a high-speed imaging technique. Hence, it could be performed using not only slow ultrasound imaging but also x-ray or magnetic resonance imaging.

Last, even if it is of high interest to retrieve the elastic modulus from the measurement of wave velocities, it is not necessarily required for diagnosis. On the basis of in vivo studies, specific ranges of wave velocities could be established, corresponding to a certain risk of cardiovascular diseases. To support the clinical usefulness of this antisymmetric wave velocity measurement, it is necessary to repeat the retinal experiment on a higher number of healthy volunteers and patients. The goal would be to establish a correlation between the FPW velocity measurement and cardiovascular diseases or risks. Using a standard clinical ultrasound scanner, the FPW velocity measurement could be easily performed. No additional material would be needed, only an ultrasonic probe to observe the carotid artery motion due to waves along the artery. In addition, there is no potential hazard because there is no need for an external shear wave source since the waves are naturally present in this artery.

In conclusion, it has been shown that a second natural pulse wave can be observed in blood vessels. It offers a reasonable explanation for the incoherent velocity estimations reported in the literature. Described as an antisymmetric wave in a tube, it is by nature highly dispersive. An analytical equation of the speed as a function of frequency and other parameters is still being developed. Present in both arteries and veins, this second natural pulse wave could be helpful in a clinical context as it is sensitive to vessel stiffness. In addition, because this wave is much slower than the standard symmetric pulse wave, an ultrafast imaging system is not necessarily needed. In particular, the standard eye fundus optic device, widely used in ophthalmology, could estimate these second pulse wave velocities as a proxy for patients’ cardiovascular health.

## MATERIALS AND METHODS

### Experimental design

#### Laser Doppler holography

LDH is a digital holographic method where blood flow is measured from the interference between coherent light backscattered by the eye and a reference beam (*26*, *27*). The coherent gain brought by the interferometric detection allows measuring the local Doppler broadening over the full-field array of an ultrafast camera with a high temporal resolution. We used the fiber Mach-Zehnder LDH setup presented in (*27*). The light source for the experiments was a 50-mW, single-mode laser diode at 785-nm wavelength (Thorlabs LP785-SAV50, VHG Wavelength-Stabilized SF Laser Diode, Internal Isolator). The power of the laser beam incident on the cornea was 3 mW of constant exposure through a beam of 1 mm by 1 mm. The retina was imaged over a field of view of approximately 5.3 mm. Interferograms were recorded using a complementary metal-oxide semiconductor camera (Ametek-Phantom V2511) operated in a 512 × 512 format at a 75-kHz frame rate. The digital holograms are first numerically reconstructed by angular spectrum propagation, and the subsequent data processing is aimed at revealing blood flow from fluctuations in the local holograms. A short-time window consisting of 2048 holograms (27.3 ms) is slid along the temporal dimension of the hologram stack. A singular value decomposition is first performed for each window to filter out the Doppler contribution due to eye motion (*28*). The pixel-wise power Doppler is then calculated as the integral of the high-pass filtered power spectrum density (squared magnitude of the temporal Fourier transform) with a cutoff frequency set to 6 kHz. Last, a correction is performed to flatten the intensity of the obtained power Doppler images, and the average image is subtracted to remove the blood flow contribution from the background.

Informed consent was obtained from all subjects, and experimental procedures adhered to the tenets of the Declaration of Helsinki. The study authorization was obtained from the appropriate local ethics review boards, Comité de Protection des Personnes(CPP) and Agence Nationale de Sécurité du Médicament (ANSM), and the clinical trial was registered under the references IDRCB 2019-A00942-5 and NCT04129021.

#### Passive spectroscopy

Passive spectroscopy algorithms are inspired by noise correlation algorithms used in passive elastography (*29*, *30*). Usually, a spatiotemporal correlation is calculated to separately measure the central wavelength and frequency, leading to the group wave velocity. Here, an original approach was used consisting in measuring the wavelength for each frequency of the bandwidth. Instead of calculating the correlation of the raw displacement signal, a Fourier transformation is first applied to the signal. Then, the correlation is computed at each frequency without considering the Fourier transform’s modulus. It allows both to normalize the correlation and to whiten the spectrum. Moreover, because the goal here was to characterize the artery globally, the normalized correlation as a function of the distance between points is averaged for all points. If the displacement signal at position *x* is written ϕ*_x_*(*r*) and its Fourier transform written $\hat{\phi_{x}}(f)$, then the focal spot at frequency *f* is calculated according to Eq. 1. The correlation profiles obtained using this technique are presented in Figs 2C and 5, C and D. A sinusoidal fit is finally performed around the central peak to measure the mean elastic wavelength. Repeating this operation at all frequencies leads to a dispersion curve for retinal arteries.

In the case of the retina, a particular approach is used. Instead of applying the algorithms to a displacement signal, the algorithms were applied to the power Doppler signal. This signal contains the information on propagating waves that are highlighted thanks to correlation algorithms. This could have never been performed using classical time-of-flight techniques.

#### Time-of-flight velocity measurements

The usual method used in shear wave elastography to measure wave velocities is called time of flight. It consists in following a wavefront across time to measure the distance traveled by the wave during a certain time. This can be done using a spatiotemporal representation of the experimental displacements and calculating the slope to retrieve the wave velocity. A similar method was implemented here to compare the results of passive spectroscopy with a more standard method. The time-of-flight technique used in this study differs from the classical method because it is based on correlation. For clarity purposes, this point is not mentioned in the main text. For a source point *x*_1_, the correlation of the displacement signal with the displacements of neighboring points is calculated, considering a time lag or not. Equation 4 details the calculation, where ⊕_Δ*t*_ is the time correlation.

$$ToF(\text{Δ}x,\text{Δ}t)={\left\langle \phi (x_{0},t){⊕}_{\text{Δ}t}\phi (x_{0}+\text{Δ}x,t)\right\rangle }_{x_{0}}$$ (4)

A linear fit on the spatiotemporal representation of the function ToF(Δx,Δt) allows for the measurement of the wave velocity. The advantage of this time-of-flight technique is that by using the correlation and the average of all source points *x*_0_, the measured wave velocity considers all the realisations of a wave propagating through the propagation medium. Hence, the signal-to-noise ratio is enhanced, and the wave velocity measurements are more accurate.

#### Numerical computation of dispersion curves

To compute the dispersion curves of the guided waves, a numerical computation of the equation derived by Gazis (*38*, *39*) in 1959 is performed. The programming language is FORTRAN90 with IMSL numerical library, and Müller’s method is used to solve for the F(1,1) and L(0,1) modes in the characteristic frequency equation established by Gazis *et al.* [equation 18 from (*38*)].

#### Ultrasound experiment

The imaging device is a 128-element L7-4 (Philips) ultrasound probe centered at 5 MHz. This probe is connected to an ultrafast ultrasound scanner (Verasonics Vantage) fully controlled using MATLAB 2017a (The MathWorks, Natick, MA, USA). The probe was placed on the left carotid of a healthy volunteer. There is no external wave source, the imaging system only records the natural motions of the carotid artery due to propagating waves. The imaging sequence comprised 1000 images acquired at a frame rate of 500 Hz. Plane waves were emitted at three different angles between +10° and −10° (*42*) to perform the compounding method (*43*). The ultrasound images were stored as in-phase and quadrature data on which phase-tracking algorithms (*44*–*46*) were used to measure the displacements of both edges of the artery as a function of space and time. The edges of the artery were manually segmented to obtain each arterial wall’s displacement signal. Then, the sum or the difference of these two displacement signals was calculated to select one or the other wave mode. Last, passive spectroscopy algorithms were applied. When comparing with the time-of-flight technique, directional filters were applied to avoid reflected waves. Furthermore, the alignment of the imaging plane with the plane of flexion of the tube could have an influence on these measurements. As long as the imaging plane is not perfectly perpendicular to the flexion plane, some displacements corresponding to the antisymmetric wave can be measured. This misalignment will affect the amplitude, but this will not affect the wavelength and thus the velocity measurement. Institutional review board approval from Inserm was acquired, and a written informed consent was obtained from all volunteers.

## Notes

View/request a protocol for this paper from *Bio-protocol*.
