# Supplementary material for: Observation of natural flexural pulse waves in retinal and carotid arteries for wall elasticity estimation
Source: Sci Adv. 2023 Jun 21;9(25):eadf1783. doi: 10.1126/sciadv.adf1783 (PMC10284541; doi:10.1126/sciadv.adf1783)
Supplement: Supplementary file 1 — Fig. S1 Legends for movies S1 to S9 [file sciadv.adf1783_sm.pdf]

Science Advances
AAAS

Supplementary Materials for

Observation of natural flexural pulse waves in retinal and carotid arteries for wall elasticity estimation

Gabrielle Laloy-Borgna *et al.*

Corresponding author: Gabrielle Laloy-Borgna, gabriellelaloyborgna@gmail.com;
Stefan Catheline, stefan.catheline@inserm.fr

Sci. Adv. **9**, eadf1783 (2023)
DOI: 10.1126/sciadv.adf1783

The PDF file includes:

Fig. S1
Legends for movies S1 to S9

Other Supplementary Material for this manuscript includes the following:

Movies S1 to S9

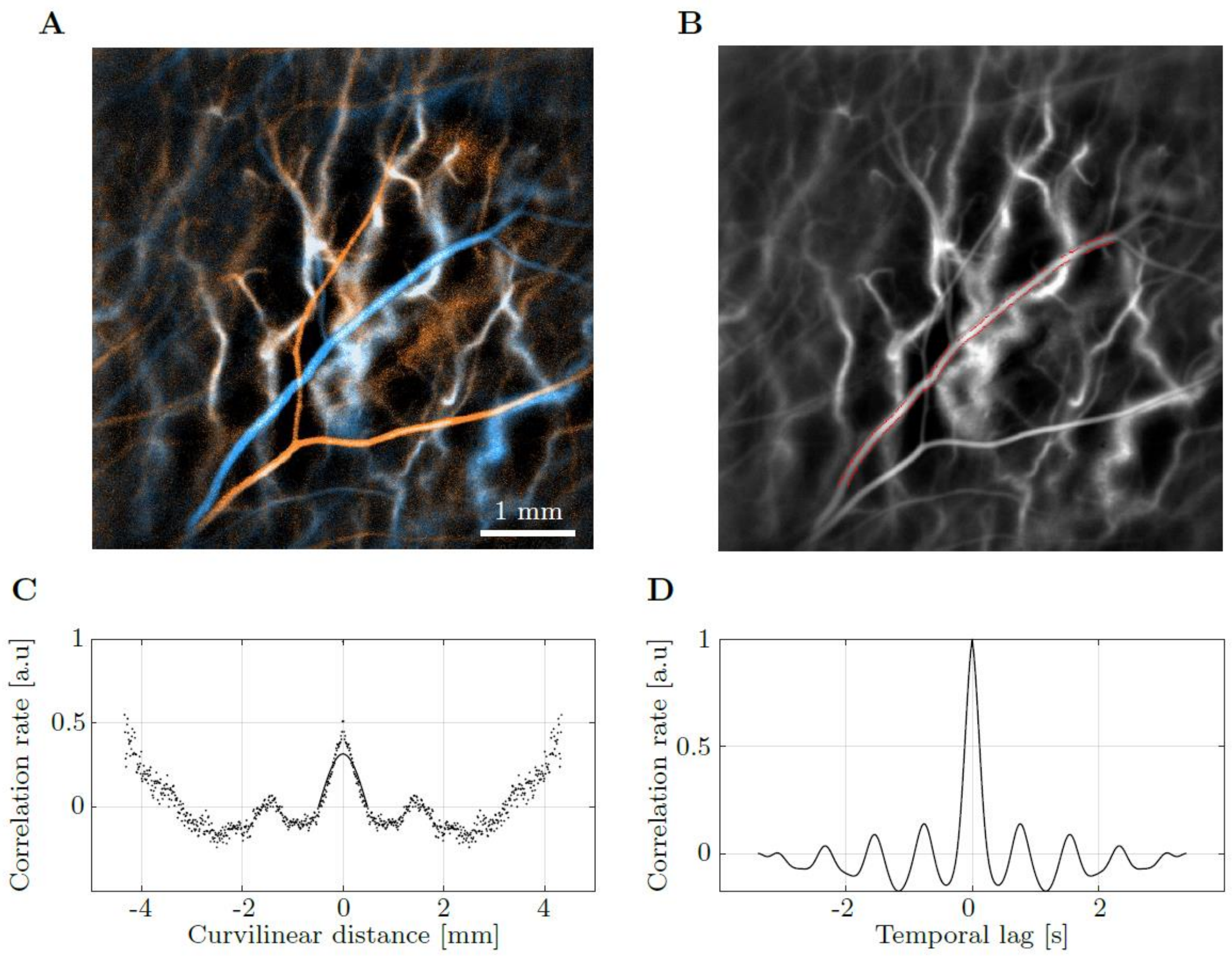

Fig. S1.

Measurement of the flexural pulse wave velocity in a vein. Arteries and veins can be distinguished thanks to the Laser Doppler holography experiments. As described in *(28)*, by fusing two images averaged from the high frequency power Doppler movie during arbitrarily chosen periods corresponding to systole and diastole, the image in orange and blue presented in Fig.S1.(a). On this type of image, arteries are colored in orange and veins in blue. In Fig.S1.(b), the result of edge detection on the main vein is shown. The Doppler signal is processed in the same way than for the arteries. The averaged focal spot allowing to measure the wavelength is shown in Fig.S1.(c), and the averaged autocorrelation is shown in Fig.S1.(d), allowing to measure the central frequency. A wavelength of 1.9 mm and a frequency of 1.31 Hz, leading to a wave velocity of 2.5 mm/s. The measurement has been repeated on three veins and three arteries of the same volunteer. The average flexural wave velocity for veins is 3.1 mm/s and 2.2 mm/s for arteries.

Movie S1. Holography movie acquired on a healthy volunteer using the experimental setup shown in Fig.1. The frame rate is 38 Hz and the field of view is $5.3 \times 5.3\ mm^2$. This is the movie used to get Fig.1 and one of the movies used to obtain Fig.3.

Movie S2. Holography movie acquired on a healthy volunteer using the experimental setup shown in Fig.1. The frame rate is 38 Hz and the field of view is $5.3 \times 5.3\ mm^2$. This is the movie used to get Fig.2 and one of the movies used to obtain Fig.3.

Movie S3. Holography movie acquired on a healthy volunteer using the experimental setup shown in Fig.1. The frame rate is 38 Hz and the field of view is $5.3 \times 5.3\ mm^2$. This is one of the movies used to obtain Fig.3.

Movie S4. Holography movie acquired on a healthy volunteer using the experimental setup shown in Fig.1. The frame rate is 38 Hz and the field of view is $5.3 \times 5.3\ mm^2$. This is one of the movies used to obtain Fig.3.

Movie S5. Displacement field of the carotid walls in arbitrary units represented as a movie. The scale bar corresponds to a distance of 5 mm and the timer shown represents the time in seconds. This movie allows to obtain Figs.4 and 5. Fig. 5(A) is frame 451 (0.902 s) and fig. 5(B) is frame 303 (0.606 s).

Movie S6. Displacement field of the carotid walls in arbitrary units represented as a movie. The scale bar corresponds to a distance of 5 mm and the timer shown represents the time in seconds. This movie corresponds to volunteer 1 in Table 1 and Fig.6.

Movie S7. Displacement field of the carotid walls in arbitrary units represented as a movie. The scale bar corresponds to a distance of 5 mm and the timer shown represents the time in seconds. This movie corresponds to volunteer 2 in Table 1 and Fig.6.

Movie S8. Displacement field of the carotid walls in arbitrary units represented as a movie. The scale bar corresponds to a distance of 5 mm and the timer shown represents the time in seconds. This movie corresponds to volunteer 3 in Table 1 and Fig.6.

Movie S9. Displacement field of the carotid walls in arbitrary units represented as a movie. The scale bar corresponds to a distance of 5 mm and the timer shown represents the time in seconds. This movie corresponds to volunteer 4 in Table 1 and Fig.6.
